# Radiotherapy for Head and Neck Cancer: Evaluation of Triggered Adaptive Replanning in Routine Practice

**DOI:** 10.3389/fonc.2020.579917

**Published:** 2020-11-12

**Authors:** Metin Figen, Didem Çolpan Öksüz, Evrim Duman, Robin Prestwich, Karen Dyker, Kate Cardale, Satiavani Ramasamy, Patrick Murray, Mehmet Şen

**Affiliations:** ^1^ Department of Radiation Oncology Şişli Hamidiye Etfal Training and Research Hospital, Istanbul, Turkey; ^2^ Department of Radiation Oncology, Cerrahpaşa Faculty of Medicine, Istanbul University-Cerrahpaşa, Istanbul, Turkey; ^3^ Department of Radiation Oncology Antalya Training and Research Hospital, Antalya, Turkey; ^4^ Department of Clinical Oncology, Leeds Cancer Center, St. James’s Institute of Oncology, Leeds, United Kingdom

**Keywords:** head and neck cancer, adaptive radiotherapy, volumetric modulated arc therapy (VMAT), replanning, chemoradiotherapy (CRT)

## Abstract

**Purpose and Objective:**

A proportion of patients receiving radiotherapy for head and neck squamous cell carcinoma (HNSCC) require *ad hoc* treatment re-planning. The aim of this retrospective study is to analyze the patients who required *ad hoc* re-planning and to identify factors, which may predict need for re-planning.

**Materials and Methods:**

A single center evaluation of all patients receiving radical or adjuvant (chemo)radiotherapy (CRT) for HNSCC between January and December 2016 was undertaken. Patients who underwent *ad hoc* re-planning during the treatment were identified in electronic records. Reasons for re-planning were categorized as: weight loss, tumor shrinkage, changes in patient position and immobilization-related factors. Potential trigger factors for adaptive radiotherapy such as patient characteristics, primary tumor site, stage, concomitant chemotherapy, weight loss ratios, radical/adjuvant treatment, and nutritional interventions were investigated.

**Results:**

31/290 (10.6%) HNSCC patients who underwent radical/adjuvant radiotherapy required re-planning. The adaptive radiotherapy (ART) was performed at a mean fraction of 15. The most common documented reasons for re-planning were tumor shrinkage (35.5%) and weight loss (35.5%). Among the patient/tumor/treatment factors, nasopharyngeal primary site (p = 0.013) and use of concurrent chemotherapy with radiotherapy (p = 0.034) were found to be significantly correlated with the need for re-planning.

**Conclusion:**

Effective on-treatment verification schedules and close follow up of patients especially with NPC primary and/or treated with concurrent chemoradiotherapy are crucial to identify patients requiring ART. We suggest an individualized triggered approach to ART rather than scheduled strategies as it is likely to be more feasible in terms of utilization of workload and resources.

## Introduction

Head and neck squamous cell carcinoma (HNSCC) is the sixth most common cancer worldwide, accounting for 6% of all cancer cases (1). Radiotherapy plays an important role in the management of HNSCC (2). Intensity-modulated radiotherapy (IMRT) and volumetric modulated arc therapy (VMAT) are the standard methods of treatment delivery for HNSCC (3). IMRT and VMAT help to reduce the dose to healthy tissue and consequently minimize the risk of toxicity. The highly conformal dose distribution allows the treatment of target volumes to therapeutic doses, potentially leading to improved loco-regional control (4, 5).

Several investigators have reported that positional, anatomical and tumor alterations can occur during radiotherapy of HNSCC. These changes may prevent the prescribed dose delivery to tumor volumes and may cause significant toxicity by increasing the dose to the organs at risk (OARs) (6–8). Adaptive radiotherapy (ART) is an approach to mitigate the dosimetry effects of these changes (9). HNSCC is the leading anatomic site in ongoing research on adaptive radiotherapy (10, 11).

Some studies noted that not all patients benefit from ART (12). Therefore, studies evaluating the need, timing and the patient selection criteria for ART have been published (13). However, there remains a lack of consensus on the implementation of re-planning in routine practice. Scheduled re-planning for ART has been investigated in some studies with limited numbers of patients (14). Some authors have suggested the use of a triggered approach to account for time dependent changes is more appropriate than scheduled ART (15). In addition to the scientific limitations, re-planning has significant departmental workload implications (16).

This study aims to evaluate the rate of re-planning and to determine the factors that predict the need for a triggered re-plan for HNSCC in routine radiotherapy practice.

## Materials and Methods

### Ethical Considerations

Institutional review board approval was obtained at St. James’s Institute of Oncology, Leeds, UK. Written informed consent for participation was not required for this study according to national legislation, since this is a retrospective audit of the standard practice.

### Patient and Disease Characteristics

A total of 290 patients older than 18 years of age with histologically proven squamous cell carcinoma of the head and neck arising from the oral cavity, oropharynx, larynx, hypopharynx, nasopharynx, sinonasal tract or unknown primary who were treated using VMAT, with or without concurrent chemotherapy, at St. James’s Institute of Oncology, Leeds, UK in 2016 were retrospectively analyzed.

The following demographics and tumor characteristics were obtained: age, gender, Karnofsky performance status scale (KPS), primary site, tumor/nodal staging, overall stage, concomitant chemotherapy status, use of induction chemotherapy, percentage weight loss (categorized as ≤5%, >5%, and >10%), treatment indication (radical or adjuvant) and feeding status (gastrostomy, use of nasogastric tube or no enteral feeding required). The impact of these factors on the decision to apply adaptive radiotherapy was evaluated.

All patients had pre-treatment computed tomography (CT) simulation. Planning CT imaging was performed on a Siemens Somatom Sensation-64 section (Siemens Healthcare, Erlangen, Germany). Patients were immobilized using a custom-fitted thermoplastic face and shoulder mask and shoulder pulls. The CT scan was performed with 2-mm-thick slices. CT data was loaded into Monaco^®^ (Elekta). The treating physician contoured the target volumes and organs at risk for each patient on the baseline planning CT scan. Planning target volume (PTV) was generated by giving a 4-mm expansion in all directions to clinical target volume (CTV). Standard radical dose was 70 Gy for high-dose PTV and 57 Gy to elective PTV. For adjuvant chemoradiotherapy (CRT) patients, a dose of 60–66 Gy was prescribed to high-risk planning target volume (PTV-HR) and 54–57 Gy to low-risk planning target volume (PTV-LR). Patients had weekly cone-beam computed tomography (CBCT). During the radiotherapy, patients were evaluated with weekly physical examination. Nutritional evaluation including weekly weight measurement before and during treatment was provided by a dietician.

The patients who required concurrent chemoradiotherapy had cisplatin 100 mg/m^2^ up to three times during treatment as the first-choice agent. Those unsuitable for this, due to comorbidity or toxicity, had weekly cisplatin (average dose 40 mg/m^2^), weekly cetuximab (except for nasopharyngeal primaries) with a loading dose of 400 mg/m^2^ and a weekly dose of 250 mg/m^2^, or carboplatin with area under the curve 4 on days 1, 22, and 43. For the patients treated with induction chemotherapy three cycles of TPF (docetaxel 75 mg/m^2^ day 1, cisplatin 75mg/m^2^ day 1, and 5-fluorouracil 750 mg/m^2^ days 2–5) was given.

### Imaging Verification and Decision Regarding On-Treatment Re-Planning

Standard imaging for treatment verification involved CBCT on days 1–3 followed by weekly CBCT. Image assessment was radiographer-led following a ‘no action level’ protocol. Proposed shifts of >3 mm mandated review by a radiotherapy physicist. The treating clinicians were contacted if there were concerns regarding target volume coverage, OAR doses or inconsistent set up. Daily CBCT was an option in the event of inconsistent set-up. Decisions regarding requirement for an adaptive re-plan were made by the clinician in conjunction with radiographers/physicists, and reasons were routinely documented as part of the process. By examining the electronic records, we highlighted the causes of decreased dose to target volumes and overdosing on OARs. Tumor shrinkage, weight loss, and positional changes (poor tongue position due to swelling, remodeling in postoperative edema, poor shoulder, or neck position) were found to be the three main reasons. A fourth reason was determined to be immobilization related factors (due to technical problems), which necessitated re-planning as a non-tumor or patient-related factor. So, we separated the reasons into four main categories. Once re-planning was required, a new mask was fabricated if there were concerns regarding mask fit. Re-planning was performed with original contours merged onto the new planning CT scan using the ABAS (Elekta) software, and these contours were checked and modified where necessary by the clinician. The clinician would make a decision whether it was acceptable to continue on the original radiotherapy plan while the new plan was being prepared. The new plan was implemented after 2–4 days.

### Statistical Analysis

Statistical analysis was performed using SPSS (IBM SPPS statistics for windows version 21.0, Armonk, NY, USA, IBM Corporation). While frequency and percentage were used for expressing categorical data, mean, standard deviation, median, lowest, and highest values were used for expressing continuous data. The Chi-square test was used to compare categorical data. The suitability of the continuous variables to normal distribution was checked by Kolmogorov Simirnov test. The Mann-Whitney U test was used to compare the means of continuous variables. Two-tailed p < 0.05 was accepted as the limit of significance.

## Results

Thirty-one (11%) patients required a re-planning CT scan. The reasons for re-planning included weight loss (35.5%), tumor shrinkage (35.5%), changes in patient position (22.5%) and immobilization-related factors (6.5%) and are summarized in **Table 1**. **Table 3** outlines the clinical and disease characteristics of the patient population treated by VMAT according to whether adaptive re-planning was utilized?

**Table 1:** Predominant indications for re-planning.

Indication	No. of patients	(%)	Radical RT N %		Adjuvant RT N %	
Weight loss	11	35.5	11	100	0	0
Primary tumor and/or nodal shrinkage	11	35.5	11	100	0	0
Changes in position	7	22.5	2	28.5	5	71.5
Immobilization related factors	2	6.5	2	100	0	0

RT, radiotherapy.

The mean age of the entire patient population was 61.3 years (range: 18–90 years) and 76% of patients were male. The most common primary tumor was oropharynx (47.9%) followed by oral cavity (16.6%), larynx (15.2%), hypopharynx (7.9%), unknown primary (6.2%), sinonasal tract (4.1%), and nasopharynx (2.1%). Using the TNM 7^th^ edition, distribution of T classification was T2 (31.7%) followed by T3 (22.8%) and T4 (22.8%). The most common N status was N2 (62.8%) and in the majority of patients, overall stage was stage-IV (74.5%) followed by stage-III (15.2%). No patient had clinical evidence of distant metastasis at diagnosis. Patient and tumor characteristics are summarized in **Table 2**.

**Table 2:** Patient, tumor, and treatment characteristics.

		Number%	
Gender	Male	221	76.2
	Female	69	23.8
Age	≤60	142	49.0
	˃60	148	51.0
Weight loss	≤%5	143	49.0
	˃%5	147	50.7
Weight loss	≤%10	248	85.5
	˃%10	42	14.5
KPS	90–100	197	67.9
	60–80	93	32.1
Nutritional intervention	No	128	44.1
	Yes	162	55.9
NG	No	253	87.2
	Yes	37	12.8
Gastrostomy	No	165	56.9
	Yes	125	43.1
Primary	Oropharynx	139	47.9
	Oral Cavity	48	16.6
	Larynx	44	15.2
	Hypopharynx	23	7.9
	Unknown primary	18	6.2
	Sinonasal	12	4.1
	Nasopharynx	6	2.1
T stage	T0	22	7.6
	T1	44	15.2
	T2	92	31.7
	T3	66	22.8
	T4	66	22.8
N stage	N0	71	24.5
	N1	30	10.3
	N2	182	62.8
	N3	7	2.4
Stage	Stage I	7	2.4
	Stage II	23	7.9
	Stage III	44	15.2
	Stage IV	216	74.5
Radiation	Radical	200	69.0
	Adjuvant	90	31.0
Chemoradiotherapy	Radiotherapy	141	48.6
	Concurrent CRT	149	51.4
Induction CT	No	276	95.2
	Yes	14	4.8

KPS, Karnofsky performance status; NG, nasogastric tube; CT, chemotherapy.

Sixty-nine % of patients underwent definitive radiotherapy and 31% of patients were treated with postoperative radiotherapy. Concurrent chemoradiotherapy was delivered in 51% of patients and 5% of patients underwent induction chemotherapy before the radiotherapy. Fifty-six % of the patients required nutritional intervention. Thirty-seven patients (12.9%) had nasogastric tube feeding and 125 patients (43.1%) gastrostomy feeding. Details are shown in **Table 2**.

Overall the rescan CT was performed at a mean fraction of 15 (range: 2–30 fractions). Only 2 patients were re-planned more than once, both were re-planned twice. One had a hypopharynx primary re-planned due to tumor shrinkage at fraction 4 and 11, the other had an oropharynx primary re-planned due to weight loss at fraction 4 and 12). The mean fraction for rescan CT was 22 (range: 3–29 fraction) for weight loss patients, 13 (range: 4–25 fractions) for tumor shrinkage patients, 10 for change in patient position (range: 2–22 fractions), and 10 for immobilization related factors.

Overall in the patient cohort (n = 290) the mean weight loss (± standard deviation) following RT was 5.0 ± 3.6 kg (range: 0 to 20.8 kg). There were no significant differences between the ART and no-ART group for any of the weight-related factors. Forty-two of 290 (14.5%) patients lost more than 10% of their body weight with only 6 of 42 patients being re-planned due to weight loss. Overall 147 of 290 (50.7%) patients lost more than 5% of their body weight with 10 of 147 being re-planned due to weight loss. One additional patient was re-planned due to weight loss less than 5% and so the total number of re-plans secondary to weight loss was 11. All of the 11 patients requiring ART due to weight loss were treated with definitive CRT. Of these, 9 underwent radiotherapy with cisplatin-based chemotherapy, 7 had oropharyngeal carcinoma, 6 were gastrostomy fed, and 1 was nasogastric (NG) tube fed. In this cohort, weight loss of more than 5% or more than 10% was not a statistically significant factor mandating ART (**Table 3**).

**Table 3:** Statistical analysis of patient and treatment related factors between no-ART and ART groups.

		No-ART		ART		p
		Number %		Number%		
Age	≤60	125	48.3	17	54.8	0.489
	˃60	134	51.7	14	45.2	
KPS	90–100	172	66.4	24	77.4	0.131
	60–80	87	33.6	7	22.6	
Weight loss	≤5%	128	49.4	15	48.4	0.913
	˃5%	131	50.6	16	51.6	
Weight loss	≤10%	224	86.5	24	77.4	0.175
	˃10%	35	13.5	7	22.6	
Nutritional interventions	No	118	45.6	10	32.3	0.171
	Yes	141	54.4	21	67.7	
NG	No	225	86.9	28	90.3	0.586
	Yes	34	13.1	3	9.7	
Gastrostomy	No	152	58.7	13	41.9	0.075
	Yes	107	41.3	18	58.1	
Primary	Oropharynx	123	47.5	16	51.6	0.807
	Oral cavity	45	17.4	3	9.7	0.404
	Larynx	42	16.2	2	6.5	0.243
	Hypopharynx	18	6.9	5	16.1	0.151
	Unknown primary	18	6.9	0	0.0	0.262
	Sinonasal Carcinoma	10	3.9	2	6.5	0.835
	Nasopharynx	3	1.2	3	9.7	***0.013***
T stage	T0	22	8.5	0	0.0	0.093
	T1	39	15.1	5	16.1	
	T2	85	32.8	7	22.6	
	T3	57	22.0	9	29.0	
	T4	56	21.6	10	32.3	
N stage	N0	66	25.5	5	16.1	0.086
	N1	29	11.2	1	3.2	
	N2	158	61.0	24	77.4	
	N3	6	2.3	1	3.2	
UICC 7^th^ ed. Stage	I	5	1.9	2	6.5	0.858
	II	21	8.1	2	6.5	
	III	41	15.8	3	9.7	
	IV	192	74.1	24	77.4	
Radiation	Radical	174	67.2	26	83.9	0.058
	Adjuvant	85	32.8	5	16.1	
CRT	Radiotherapy	132	51.0	9	29.0	***0.034***
	Concurrent CRT	127	49.0	22	71.0	
Induction CT	No	248	95.8	28	90.3	0.179
	Yes	11	4.2	3	9.7	
^m^Mann–Whitney u test/^X²^ Chi-square test						

KPS, Karnofsky performance status; NG, nasogastric tube; CT, chemotherapy; CRT, chemoradiotherapy. Statistically significant values are in bold/italic.

All the patients who underwent re-planning due to tumor shrinkage were treated with definitive radiotherapy with 9 of 11 patients treated with concurrent chemoradiotherapy. The tumor shrinkage group consisted of 5 oropharyngeal, 3 hypopharyngeal, 2 nasopharyngeal, and 1 laryngeal primary. The majority of patients had stage IV disease (82%).

Change in patient position was the third cause of ART, with 5 of 7 cases occurring in the adjuvant radiotherapy group. Of these patients 3 had an oral cavity primary and resolution of postoperative edema was the reason for positional change. Only 2 patients required re-planning for mask fit related reasons.

Having a nasopharyngeal primary was a statistically significant risk factor for re-planning (p = 0.013) however there were only six patients with nasopharyngeal tumor in this study. In total, 3 out of 6 patients with a nasopharyngeal primary required ART – 2 of these secondary to tumor shrinkage and 1 secondary to weight loss of more than 10%. All three cases received chemotherapy in addition to radiotherapy (2 had cisplatin 100 mg/m^2^ and 1 had carboplatin with previous induction chemotherapy). The mean fraction for rescan CT was 21 (range, 10–29). No other primaries were found to be correlated with need for ART (**Table 3**).

More patients in the ART group received concurrent chemoradiotherapy compared to the no-ART (71% versus 49% of patients) and concurrent chemotherapy was an independent risk factor for need for ART (p = 0.034). There was however no statistically significant difference between chemotherapy regimens.

Of those requiring ART 83.9% were being treated with radical intent, in the non-ART group the radically treated patients accounted for 67.2%, as shown in **Table 3**. Definitive intent treatment had a trend towards need for ART but a statistically significant difference was not encountered (p > 0.05).

There was no significant difference in the distribution of gender, age, stage, KPS, nutrition intervention (Gastrostomy or NG) or use of induction chemotherapy between the ART and no-ART groups.

## Discussion

Head and neck cancers are subject to significant variations due to tumor shrinkage, weight loss and positional changes during radiotherapy. ART is a strategy to correct these changes to minimize detrimental effects and potentially increase tumor control. However, there are clinical and technical challenges with the routine use of ART. Due to additional workload and economic factors it is important to identify patients most likely need or benefit from re-planning.

Historical publications focused on dosimetric impact of anatomical changes during the course of radiotherapy. Hansen et al. demonstrated an increase in D max (maximum dose) to spinal cord and brainstem during radiotherapy (9). Several investigators reported significantly increased dose delivery to parotid glands during the course of radiotherapy (17, 18). More recent studies have focused on the necessity of ART and a survival advantage associated with ART has been shown in some studies (19, 20). Yang et al. achieved superior 2-year loco-regional control and improvement in quality of life with ART (20). Zhao L. et al. found improved relapse-free survival for T3 and T4 nasopharyngeal carcinoma (NPC) with ART (21). Chen et al. stated that 317 HNSCC patients underwent IMRT and 51 out of 317 had midway re-planning. They reported superior 2-year local control with ART (88 vs. 79%; p < 0.05) (22). However, it is difficult to determine whether the survival advantage is due to ART or whether those requiring ART have more favorable tumor biology with early response. It has been shown, that early regression of gross tumor volume is correlated with higher complete metabolic response rates and with improved PFS and local control rates (23–25). The debate about the positive effect of ART on survival remains uncertain.

There is limited data to identify the candidates most likely to benefit from ART (11, 26, 27). Brown et al. stated having more advanced nodal disease, nasopharyngeal primary, being treated with tomotherapy and VMAT compared to IMRT are the factors contributing to re-planning (11). Sürücü et al. found using standard cisplatin rather than low-dose cisplatin or cetuximab and being younger than 47 were predictive for increased primary tumor volume reduction (26). Yu Chang Hu et al. reported superior PTV coverage with re-planning for nasopharyngeal primary patients with a BMI of >21.5, initial weight (>60 kg), obvious weight loss (>2.8kg), concurrent chemoradiotherapy and stages III-IV disease (27).

Weight loss is a common problem in HNSCC patients during CRT. The literature provides contradictory data about the correlation of weight loss and the necessity for ART. Wei-Hsien Hou et al. found that NPC patients with weight loss > 5% and smaller PTVs were more likely to have increased setup errors (28). Mahmoud et al. found weight loss of more than 9.6% as the critical value to trigger ART in terms of target under dosing and/or spinal cord/parotids overdosing (29). The study by Real et al. showed that patients losing more than 5% of weight underwent higher rates of ART due to shifts of the PTV and parotid gland (30). By contrast, Noble et al. noted that a median loss of 7.9% weight was not a trigger for re-planning in terms of spinal cord safety (31). Kean Fat Ho et al. found losing more than 10% weight did not affect dose delivery to OARs and did not prompt ART (32). So far, there is no consensus regarding the cut-off value of weight loss prompting ART. We believe that one of the main reasons for this is the fact that there is no agreed single end point for risk from weight loss. Inadequate PTV coverage and excess dose to spinal cord/parotid are all identified as potential outcomes of changes in dosimetry resulting from weight loss. In our series, 42 out of 290 patients lost more than 10% of their body weight and 7 out of 42 need re-planning. There was however no statistically significant correlation with re-planning and weight loss more than 10%. However, all the patients having ART due to weight loss were treated curatively with definitive (chemo)radiotherapy, similar to literature. Although all the patients underwent weekly intensive nutritional counselling by a dietician and feeding intervention ratio in the overall cohort was high (55.8%), this did not prevent 14.5% of patients experiencing weight loss of > 10%. 21 out of the 31 patients who required a re-plan also required a feeding intervention and in 7 of these the trigger for ART was weight loss itself. This highlights that even in the setting of feeding interventions such as gastrostomy or NG, patients continue to have the potential for weight-loss related dose deviation.

ART is mainly utilized and advocated for locally advanced HNSCC patients, especially those who are treated with definitive chemoradiotherapy (14, 26, 33, 34). In the present series and in line with current literature, patients who underwent chemoradiotherapy had a statistically significant higher re-plan rate compared to sole radiotherapy. In addition, re-planning was more frequent in patients with an advanced T (T3-T4) and N (N2-N3) stage.

Nasopharyngeal primaries are generally diagnosed in the locally advanced stage and primary treatment is definitive chemoradiotherapy (35). All the patients with NPC in our series had locally advanced disease. Although, the number of NPC is limited in this study, we observed a significant increase of ART in NPC primaries compared to other primaries (p < 0.05). Half of the NPC patients required ART.

Definitive intent patients had a higher rate of re-planning compared to postoperative patients although this did not reach statistical significance (p = 0.058). There was no statistically significant difference between adjuvant and definitive intent groups. Five out of 90 adjuvant intent patients had ART. Three patients with an oral cavity tumor required ART due to the resolution of postoperative edema and/or remodeling of the surgical graft with a mean day of 15 days (range, 9–22). This supports the recommendation of Chen et al. (22) regarding close follow-up of the surgical graft and wound site for oral cavity patients during the course of CRT in relation to the need for a re-plan.

The optimal timing of ART is not well defined in the literature. Bhide et al. stated that the maximum rate of shrinkage in tumor volume occurred at week 2 (36). In contrast, Barker et al. identified a 1.8% reduction in tumor size per treatment day with the greatest dosimetric impact occurring at week 3 or 4 and so proposed re-planning at this interval (6). Yang et al. differed proposing re-planning at week 4 or 5 (20) and Ahn et al. proposed a wider range for ART between fractions 11 to 33 (12). Overall well-defined timing for ART in HNSCC has not been identified.

NPC is the leading condition for ongoing ART research in head and neck cancers. Despite this, there remains no consensus regarding ART in NPC. Wang et al. suggested that patients diagnosed with a NPC have a significant benefit if re-planning is done before the 25^th^ fraction (37). Danfang Yan et al. determined the most appropriate time for re-planning of NPC was after the 20^th^ fraction (38). Brown et al. recommended a re-plan CT at the start of week three (13). By contrast Fung et al. proposed 3 re-plans at 9^th^, 19^th^ and 29^th^ fraction for NPC (39). In our series average day for re-planning CT was fraction 15 (range, 2–30) for overall HNSCC and fraction 21 (range, 10–29) for NPC patients. We observe that the wide range in our data is comparable with the discrepancy regarding timing of ART in the literature. Two studies based on a triggered ART strategy identified fraction 16 and fraction 15 as the mean time for re-planning consistent with our current findings (19, 21). Overall it is not possible to determine an optimal time frame for ART due to heterogeneity in patient groups and different reasons prompting ART. More individualized strategies must be developed and as such a triggered approach rather than a scheduled approach might be more useful. In line with this recently Van Beek et al. published their ‘traffic light action protocol’ to cope with changes during HNSCC RT (33).

Up to now, a limited subset of patients gets benefit from ART (12, 40). Furthermore, even if the patients get a dosimetric benefit from ART this might have no clinical impact for some patients (41).

The present study was limited by the retrospective nature of the analysis. The distribution of patients was not homogeneous. Selection was done at the discretion of the treating physician with no defined protocol. This inter observer variability might have led to differences in patient choices for ART. Analyzing definitive and adjuvant patients in the same group for predictive factors may be problematic and this was done to highlight the use of ART in routine clinical practice.

In conclusion, it is crucial to identify the patients who will potentially benefit from ART as it is a time and resource-consuming intervention. According to our findings, potential factors associated with higher rates of re-planning in HNSCC patients are nasopharyngeal primary and concurrent administration of chemotherapy with radiotherapy. However, it may never be possible to predict a distinct optimal time scale. We suggest an individualized triggered approach to ART rather than scheduled strategies. This approach is also likely to be more feasible in terms of workload and resources. Close monitoring of the patient and a multidisciplinary approach with good collaboration amongst clinicians and allied health professionals is essential in terms of detecting the need for ART during a patient’s treatment course. In the future, in the era of online adaptive radiotherapy, individualized strategies should therefore be pursued.

## Data Availability Statement

The original contributions presented in the study are included in the article/supplementary materials. Further inquiries can be directed to the corresponding author/s.

## Ethics Statement

Institutional review board approval was obtained at St. James’s Institute of Oncology, Leeds, UK. Written informed consent for participation was not required for this study since this is a retrospective audit of the standard practice.

## Author Contributions

MF, DÖ, and MŞ Conceptualized and designed the study. RP edited the manuscript. RP, KD, KC, SR, PM, ED, and MŞ: collected the data. All authors contributed to the article and approved the submitted version.

## Conflict of Interest

The authors declare that the research was conducted in the absence of any commercial or financial relationships that could be construed as a potential conflict of interest.

This study was presented as poster in part at the 7th ICHNO International Congress on Innovative Approaches in Head & Neck Oncology 14–16 March 2019 in Barcelona.
